# Disproportionate Incidence of COVID-19 Infection, Hospitalizations, and Deaths Among Persons Identifying as Hispanic or Latino — Denver, Colorado March–October 2020

**DOI:** 10.15585/mmwr.mm6948a3

**Published:** 2020-12-04

**Authors:** Laura Jean Podewils, Tori L. Burket, Christie Mettenbrink, Abigail Steiner, Allison Seidel, Kenneth Scott, Lilia Cervantes, Romana Hasnain-Wynia

**Affiliations:** ^1^Denver Public Health, Denver Health and Hospital Authority, Denver, Colorado; ^2^Office of Research, Denver Health and Hospital Authority, Denver, Colorado; ^3^School of Public Health, University of Colorado Anschutz Medical Campus, Denver, Colorado; ^4^Department of Medicine, Denver Health and Hospital Authority, Denver, Colorado; ^5^Department of Medicine, University of Colorado Anschutz Medical Campus, Denver, Colorado.

Persons identifying as Hispanic or Latino (Hispanic) represent the second largest racial/ethnic group in the United States (*1*), yet understanding of the impact of coronavirus disease 2019 (COVID-19) in this population is limited. To evaluate COVID-19 health disparities in the community and inform public health, health system, and community-based interventions, local public health authorities analyzed the sociodemographic characteristics of persons who were diagnosed, hospitalized, and who died with COVID-19 in Denver, Colorado. During the first 7 months of the COVID-19 epidemic in Denver (March 6–October 6, 2020) the majority of adult COVID-19 cases (54.8%), hospitalizations (62.1%), and deaths (51.2%) were among persons identifying as Hispanic, more than double the proportion of Hispanic adults in the Denver community (24.9%) (*1*). Systemic drivers that influence how Hispanic persons live and work increase their exposure risks: compared with non-Hispanic persons, Hispanic persons with COVID-19 in Denver reported larger household sizes and were more likely to report known exposures to household and close contacts with COVID-19, working in an essential industry, and working while ill. Reducing the disproportionate incidence of COVID-19 morbidity and mortality among Hispanic persons will require implementation of strategies that address upstream social and environmental factors that contribute to an increased risk for both infection and transmission and that facilitate improved access to culturally congruent care.

Staff members from Denver Public Health, a department of Denver Health and Hospital Authority (DHHA), conducted interviews or reviewed medical records for all persons with diagnosed laboratory-confirmed COVID-19 who resided in the city and county of Denver per notification of the Colorado Electronic Disease Reporting System* during March–October 2020. Interviews with persons whose primary language was Spanish were conducted in Spanish by bilingual interviewers or through the DHHA language line, which provides 24/7 access to professional interpreters for over 240 languages. Staff members gathered sociodemographic and epidemiologic information, including potential sources of exposure (e.g., household, close contact, and recent travel), signs and symptoms, symptom onset date, and whether the respondents worked while ill. In early May, the interview form was expanded to include detailed information on industry and occupation according to national guidelines (*2*) and household size. Because of the large volume of cases and difficulty reaching persons in the hospital, medical chart reviews, rather than telephone interviews, were used to obtain information about persons hospitalized or deceased at the time of COVID-19 diagnosis. Data from case interviews and medical chart reviews were obtained from standardized case report forms, validated for completeness, and entered into a secure REDCap database (*3*). The analysis used public health surveillance data and was carried out to understand and inform public health actions to control the spread of COVID-19 in the Denver community; the project was determined to be nonhuman subjects’ research and exempt by the Colorado Multiple Institutional Review Board.

The analysis focused on adults aged ≥18 years living in noncongregate settings (excluding persons in long-term care facilities, jails, or in shelters for persons experiencing homelessness) at the time of diagnosis and aimed to identify COVID-19 health disparities in the community to inform public health, health system, and community-based interventions. The proportions of adults with laboratory-confirmed COVID-19, those who were hospitalized for COVID-19, and the proportion of persons with COVID-19 who died were assessed by age, sex, and race/ethnicity. Additional analyses, for each COVID-19 cases and hospitalized patients, focused on comparisons between persons who identified as Hispanic to those who identified as non-Hispanic (all other racial/ethnic groups combined) to assess differences in sociodemographic characteristics, source of COVID-19 exposure, symptoms, occupation, whether they worked while ill, and household size. Occupational industry codes were categorized as essential or nonessential according to a framework developed for Colorado.^†^ T-tests and Mann-Whitney tests were used to compare continuous variables, and chi-squared tests were used for categorical variables to determine differences between racial/ethnic groups; an alpha level of 0.05 was used to determine statistical significance. All analyses were conducted in Stata (version 15.0; StataCorp).

The first event of laboratory-confirmed COVID-19 in a Denver resident was reported on March 6, 2020. During the first 7 months of the epidemic in Denver (March 6–October 6), COVID-19 was diagnosed in 10,163 adults living in noncongregate settings, including 1,087 (10.7%) persons who were hospitalized at the time of diagnosis and 165 (1.6%) who died during this period.

The highest proportions of infection occurred among persons aged 25–44 (49.1%) and 45–64 (26.6%) years (Table 1). Race and ethnicity data were available for 9,056 (89.1%) persons with diagnosed COVID-19. A total of 4,959 (54.8%) of persons diagnosed with COVID-19 in Denver occurred among Hispanic persons, approximately double the proportion of adults in Denver identifying as Hispanic (24.9%) (*1*). In contrast, 32.3% of persons diagnosed with COVID-19 identified as non-Hispanic White (White), and 6.4% identified as non-Hispanic Black or African American (Black), subpopulations that constitute 56.8% and 8.5%, respectively, of Denver adults. The pandemic’s initial surge (March 1–June 14, 2020) included more cases and persisted longer among persons of Hispanic ethnicity compared with those of other racial/ethnic groups (Figure). During subsequent surges (June 14–September 5 and September 6–October 3), patterns among Hispanic and White persons were similar, with consistently higher numbers among Hispanic persons.

**TABLE 1:** Sociodemographic characteristics of adults aged ≥18 years* with laboratory-confirmed COVID-19 — Denver, Colorado, March 6, 2020–October 6, 2020

Characteristic (no. with available information)	No. (%)^†^		
	Cases (n = 10,163)	Hospitalizations (n = 1,087)	Deaths (n = 165)
**Age group, yrs (10,163)**			
18–24	1,621 (16.0)	37 (3.4)	3 (1.8)
25–44	4,990 (49.1)	245 (22.5)	9 (5.5)
45–64	2,704 (26.6)	462 (42.5)	55 (33.3)
≥65	848 (8.3)	343 (31.6)	98 (59.4)
**Sex (10,163)**			
Men	4,851 (47.7)	566 (52.1)	106 (64.2)
Women	5,312 (52.3)	521 (47.9)	59 (35.8)
**Race/Ethnicity^§^ (9,056)**			
White	2,926 (32.3)	167 (18.2)	36 (29.8)
Black or African American	579 (6.4)	105 (11.5)	14 (11.6)
Hispanic	4,959 (54.8)	569 (62.1)	62 (51.2)
Asian	315 (3.5)	36 (3.9)	3 (2.5)
American Indian/Alaska Native	51 (0.6)	8 (0.9)	1 (0.8)
Native Hawaiian/Pacific Islander	47 (0.5)	6 (0.7)	1 (0.8)
Other/Mixed race	179 (2.0)	26 (2.8)	4 (3.3)

**Abbreviation:** COVID-19 = coronavirus disease 2019.

* In noncongregate living situations.

**^†^** Percentages reflect the proportion of persons with nonmissing values for the indicator; race/ethnicity information was available for 9,056 (89.1%) persons.

^§^ Racial/ethnic categories are mutually exclusive. Hispanic persons could be of any race; other racial/ethnic groups were non-Hispanic (e.g., White = non-Hispanic White). The Other/Mixed race category included persons who identified as two or more different races or who did not identify by the listed race categories or as Hispanic (e.g., Burmese, Egyptian, or Filipino).

**FIGURE:**
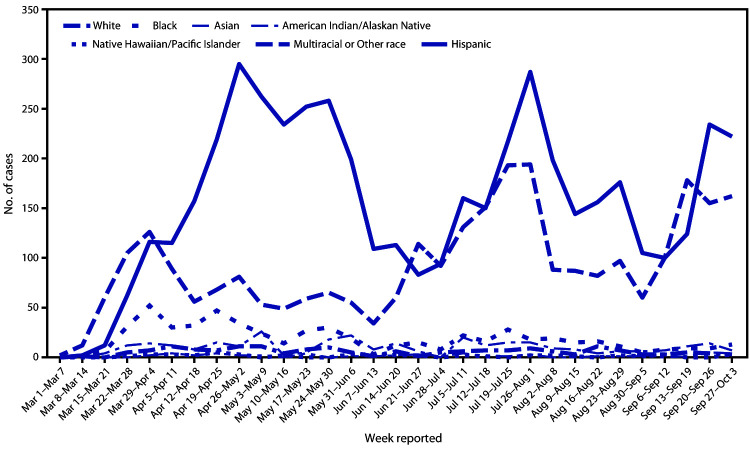
Adult COVID-19 cases, by race/ethnicity and reported week — Denver, Colorado, March 01–October 03, 2020* **Abbreviation:** COVID-19 = coronavirus disease 2019. * Only full weeks are depicted in figure during evaluation period.

Hispanic persons accounted for 62.1% of hospitalizations and 51.2% of deaths (Table 1). Whereas Hispanic adults with COVID-19 overall were slightly older than non-Hispanic adults (mean age = 40.8 years versus 39.6 years) (p<0.001), Hispanic adults who were hospitalized with COVID-19 were significantly younger than non-Hispanic adults (mean age = 52.8 years versus 60.2 years) (p<0.001) (Table 2). The distribution of cases was similar among males and females in both Hispanic and non-Hispanic adults. Approximately 90% of both Hispanic and non-Hispanic cases reported symptoms, but Hispanic persons with COVID-19 were significantly more likely than were non-Hispanic persons to report symptoms (p<0.001). Among those who were symptomatic, the median interval between symptom onset and specimen collection was 4 days among Hispanic adults compared with 3 days among non-Hispanic adults (p<0.001). The proportions of Hispanic and non-Hispanic persons who reported experiencing cough, shortness of breath, fatigue, headaches, or diarrhea were similar; however, symptomatic Hispanic patients reported a higher number of total known COVID-19 symptoms (p<0.001) (Table 2). Persons who identified as Hispanic, compared with non-Hispanic, were significantly more likely to report fever or chills (52.7% versus 48.4%; p = 0.03), muscle aches (54.1% versus 48.3%; p<0.001), loss of taste or smell (28.7% versus 22.9%; p<0.001), and a sore throat (34.7% versus 30.7%; p = 0.005).

**TABLE 2:** Sociodemographic and clinical characteristics of adults with laboratory-confirmed COVID-19 and hospitalized COVID-19 patients, by Hispanic ethnicity — Denver Colorado March 6–October 6, 2020

Characteristic	No (%)*		p-value
	Non-Hispanic	Hispanic	
**Cases (N = 9,056)**			
**No. (% of total cases)**	**4,097 (45.2)**	**4,959 (54.8)**	—
**Mean age (SD), yrs**	39.6 (0.3)	40.8 (0.2)	<0.001
**Age group, yrs**			
18–24	698 (17.0)	771 (15.5)	<0.001
25–44	2,127 (51.9)	2,339 (47.2)	
45–64	882 (21.5)	1,532 (30.9)	
≥65	390 (9.5)	317 (6.4)	
**Sex**			
Men	1,979 (48.3)	2,309 (46.6)	0.10
Women	2,118 (51.7)	2,650 (53.4)	
**Symptomatic**			
No	325 (9.0)	249 (5.7)	<0.001
Yes	3,272 (90.7)	4,134 (94.3)	
**Days from symptom onset to laboratory test (symptomatic cases only)**			
Median (IQR)	3 (1,6)	4 (2,7)	<0.001
**No. of known COVID-19 symptoms^†^ at diagnosis (symptomatic cases only)**			
1	360 (11.5)	346 (8.7)	<0.001
2–4	1,499 (47.8)	1,924 (48.3)	
5–6	952 (30.3)	1,191 (29.9)	
>6	328 (10.4)	523 (13.1)	
**Worked while ill (symptomatic cases only)**			
No	325 (22.7)	249 (13.6)	<0.001
Yes	1,110 (77.3)	1,585 (86.4)	
**Work in essential industry^§^**			
No	476 (39.8)	558 (31.2)	<0.001
Yes	719 (60.2)	1,229 (68.8)	
**No. of persons in household^¶^**			
1 (lives alone)	345 (19.6)	138 (6.4)	<0.001
2	657 (37.3)	387 (18.0)	
3–4	534 (29.7)	803 (37.3)	
5–6	173 (9.8)	599 (27.8)	
>6	64 (3.6)	227 (10.5)	
**Source of exposure: known household contact**			
No	3,474 (84.8)	3,785 (76.3)	<0.001
Yes	623 (15.2)	1,174 (23.7)	
**Source of exposure: close contact**			
No	3,371 (82.3)	4,159 (83.9)	0.04
Yes	726 (17.7)	800 (16.1)	
**Source of exposure: household and close contact**			
No	4,027 (98.3)	4,840 (97.6)	0.02
Yes	70 (1.7)	119 (2.4)	
**Hospitalizations (N = 917)**			
**No. (% of total hospitalizations)**	**348 (38.0)**	**569 (62.1)**	<0.001
**Mean age (SD)**	60.2 (0.9)	52.8 (0.7)	
**Age group, yrs**			
18–24	8 (2.3)	26 (4.6)	<0.001
25–44	69 (19.8)	157 (27.6)	
45–64	131 (37.6)	251 (44.1)	
≥65	140 (40.2)	135 (23.7)	
**Sex**			
Men	185 (53.2)	292 (51.3)	0.59
Women	163 (46.8)	277 (48.7)	

**Abbreviations:** COVID-19 = coronavirus disease 2019; IQR = interquartile range; SD = standard deviation.

*Race and ethnicity data available for 9,056 of 10,163 cases (89.1%) and 917 of 1,087 (84.4%) hospitalizations. Percentages reflect proportion of persons with non-missing values for the indicator.

^†^ Known COVID-19 symptoms include fever or chills, cough, shortness of breath, fatigue, muscle aches, headache, new loss of taste or smell, sore throat, nausea or vomiting, and diarrhea. Range = 1–9.

^§^ Detailed information on employment was only obtained on a subset of cases (n = 2,982, 33%), as collection of this information began later in the epidemic. Specified proportions of workers in each of the following 10 sectors are considered essential in Colorado: agriculture, forestry, fishing and hunting (100%); mining (100%); construction (100%); manufacturing (100%); wholesale trade (100%); retail trade (60%); transportation, warehousing, and utilities (100%); waste management (18%); education, health care and social assistance (100%); food services (64%); other services, including auto repair, child care, banks, and laundries (40%).

^¶^ Household size was only available for a subset of cases (n = 3,917, 43%), because this field was introduced later in the epidemic as obtaining information on close contacts for contact tracing became part of standard case interviews.

A higher percentage of symptomatic Hispanic persons with COVID-19 reported working while ill (86.4%) than did non-Hispanic persons with COVID-19 (77.3%; p<0.001). Among the subset of 2,982 (32.9%) persons with detailed employment information available, 68.8% of Hispanic adults reported working in essential industries compared with 60.2% of non-Hispanic adults (p<0.001). Among 3,917 (39.0%) persons with COVID-19 who provided information about household contacts, 38.3% of Hispanic persons reported five or more persons in the household, compared with 13.4% of non-Hispanic persons reporting the same (p<0.001). In addition, reported exposure to a person with known COVID-19 in the household was significantly higher among persons who identified as Hispanic (23.7%) than among those who identified as non-Hispanic (15.2%), as was reporting both exposure within the household and close contact outside the household with a person with known COVID-19 (2.4% versus 1.7%, respectively; p<0.02).

## Discussion

These findings indicate that COVID-19 has disproportionately affected Hispanic persons in the Denver community. Overall, the proportions of COVID-19 cases, hospitalizations, and deaths among Hispanic adults were approximately double the proportion of Hispanic adults in the Denver community. A recent study in Connecticut did not identify significant disparities between persons identifying as Hispanic and those identifying as non-Hispanic, but race/ethnicity data were missing for >55% of cases (*4*); in contrast, race/ethnicity data were available for >89% of patients in the current study. These findings are similar to national data reporting that Hispanic persons have approximately twice the likelihood of serious COVID-19 or death compared with White persons (*5*). This analysis provides a more comprehensive picture of COVID-19 disparities in the Denver community than has been previously available.

Although a higher prevalence of underlying health conditions (e.g., diabetes and obesity) among persons who identify as Hispanic^§^ might increase risk for severe disease, cultural and socioeconomic factors related to how persons live and work influence COVID-19 exposure, incidence, and clinical course. Denver adults with COVID-19 who identified as Hispanic were more likely to be members of larger households, to have known exposure to persons with COVID-19, to work in essential industries, and to continue to work while ill, than were those with COVID-19 who identified as non-Hispanic. Whereas social networks among Hispanic persons living in the United States are often viewed as protective for chronic health conditions (*6*), in the case of a readily transmissible infectious disease without any known immunity, such as COVID-19, close networks present elevated risk for exposure and infection. The data from this study show that Hispanic persons in Denver disproportionately work in essential industries such as agriculture, construction, health care, food services, and waste management, where workers might continue working while ill because of economic concerns or lack of paid medical leave (*7*,*8*). In addition, Hispanic adults were more likely to report symptoms and have symptoms for 1 day longer than were non-Hispanic adults before seeking laboratory testing, which might reflect barriers related to testing and health care access (*8*).

The findings in this report are subject to at least five limitations. First, information was obtained at the time of the case report, and limited information was available on outcomes after the interview. Second, data for patients who could not be contacted, who were hospitalized, or who had died were gathered through electronic medical records, which might not be as comprehensive as are interviews. Third, the interview form underwent multiple iterations to better respond to the evolving epidemic; thus, information on employment and household size was not available for all cases. Fourth, persons were categorized as Hispanic or non-Hispanic for the majority of comparisons examining sociodemographic and clinical factors after the initial comparison across different race/ethnicity categories revealed the majority of the incidence among Hispanic persons. Persons of Hispanic ethnicity are not a homogenous group, and this aggregation did not allow for further examination by racial category among the Hispanic population. Finally, because of the need for Denver Public Health to serve as a trusted support for persons with COVID-19, information on immigration status was not solicited. However, Hispanic immigrants might be more likely to hold jobs that do not include paid medical leave and might have limited access to health care, resulting in seeking health care later and poorer outcomes (*7*,*8*).

In this study of COVID-19 cases in Denver, Hispanic persons were at increased risk for acquiring COVID-19, which might be partially attributable to frequent household and workplace exposure and for COVID-19–associated hospitalization and death. A constellation of community, system, and individual factors, including systemic discrimination, likely lead to health inequalities that have been amplified by the COVID-19 epidemic. Public health and clinical health systems have opportunities and obligations to address health inequities in the communities they serve. Because several factors leading to disproportionate exposure, such as crowded housing and lack of paid medical leave, are attributable to upstream social drivers and outside the traditional health care system, public health and health care systems should partner with social service organizations and community health workers to address patients’ unmet social, medical, and mental health needs while providing culturally congruent prevention information on COVID-19 (*9*).

**Summary**

**What is already known about this topic?**

Racial and ethnic disparities of COVID-19 have been noted at the national level, but community-level data are limited.

**What is added by this report?**

In Denver, Colorado, the majority of adult COVID-19 cases (55%), hospitalizations (62%), and deaths (51%) were among Hispanic adults, double the proportion of Hispanic adults in Denver (24.9%). Among adults with COVID-19, Hispanic persons reported larger household sizes and more known COVID-19 household exposure, working in essential industries, working while ill, and delays in testing after symptom onset.

**What are the implications for public health practice?**

Public health, health systems, and social services need to address systemic inequalities to mitigate the disproportionate incidence of COVID-19 in Hispanic persons.

## Footnotes

*
https://www.colorado.gov/pacific/cdphe/report-a-disease .

† https://www.coloradohealthinstitute.org/research/colorado-covid-19-social-distancing-index.

§ https://nccd.cdc.gov/weat/#/crossTabulation/viewReport.
